# Navigating regulatory and policy challenges for AI enabled combination devices

**DOI:** 10.3389/fmedt.2024.1473350

**Published:** 2024-11-28

**Authors:** Snigdha Santra, Preet Kukreja, Kinshuk Saxena, Sanyam Gandhi, Om V. Singh

**Affiliations:** ^1^ Chugai Pharma, Berkeley, NJ, United States; ^2^ Department of Population Health, Episcopal Health Services, New York, NY, United States; ^3^ Business Strategy Department, Novartis Pharmaceuticals, Hanover, NJ, United States; ^4^ Takeda Research Center, Cambridge, MA, United States; ^5^ Advance Academic Programs, Krieger School of Arts and Sciences, Johns Hopkins University, Washington, DC, United States

**Keywords:** AI-enabled combination devices, AI regulatory frameworks, FDA AI regulations, AI-enabled medical devices, AI policy frameworks, AI regulatory challenges

## Abstract

In recent years, the Artificial Intelligence (AI) has enabled conventional Combination Devices (CDs) to innovate in healthcare merging with technology sectors. However, the challenges like reliance on predicate devices in US Food and Drug Administration (FDA's 510(k) pathway, especially for perpetually updating AI are stressed. Though the European Union (EU's new Medical Device Regulations address software and AI, fitting adaptive algorithms into conformity assessments remains difficult. The urgent need for frameworks cognizant of AI risks like model degradation and data biases is emphasized. Insights from recalled devices and case studies elucidate challenges in regulatory navigation for manufacturers. Adaptive policy frameworks balancing patient safeguards and rapid innovation are proposed. Recommendations target regulators and policy makers, advocating global standards to enable safe, effective and equitable AI adoption. This article aims to examine AI-enabled combination device regulation, inspecting US and EU strategies as well as obstacles for manufacturers and regulators.

## Introduction

Artificial intelligence (AI)-enabled Combination Devices (CD), which combine AI with conventional medical devices to improve their functionality, accuracy, and adaptability, represent a significant advancement in the healthcare and technology sectors. AI-enabled CDs differ from conventional medical devices in their ability to learn from data, adapt their performance over time, and make autonomous or semi-autonomous decisions. Common AI technologies in CDs include machine learning algorithms for image analysis in diagnostic devices, natural language processing in patient monitoring systems, and reinforcement learning in drug delivery devices. These are applied in areas such as personalized insulin delivery systems, AI-assisted surgical robots, and smart cardiac monitoring devices. While offering the potential to enhance patient outcomes, the incorporation of AI into medical devices also presents challenges for legislators and regulatory agencies in terms of data privacy, algorithmic transparency, and ensuring patient safety.

Legislators and regulatory bodies face challenges when AI is incorporated into medical devices, primarily in the areas of data privacy, algorithmic transparency, and patient safety. Strong security measures are required to address data privacy issues in order to stop unauthorized access and unethical use of patient data (1, 2). To maintain confidence, algorithmic transparency is necessary, mandating precise documentation and justifications of AI decision-making procedures (3). In order to receive commercial certification, AI-enabled medical devices have to clear stringent testing and validation processes to ensure patient safety and efficacy (4).

Regulatory frameworks specifically designed for CDs have been developed by the European Commission in the European Union (EU) and the Food and Drug Administration (FDA) in the US. Because AI technologies are constantly evolving, the FD's proposed framework for AI/ML-based Software as a Medical Device (SaMD) places a strong emphasis on striking a balance between pre-market assessment for large changes and a streamlined approach for minor alterations (5). To guarantee high safety and performance standards, in contrast, the E's Medical Devices Regulation (MDR) and *in vitro* Diagnostic Regulation (IVDR) establish strict pre-market and post-market procedures (6). In order to fully explore the potential of AI-enabled combination devices in healthcare, a flexible and well-informed regulatory and policy approach that prioritizes safety, efficacy, and ethical issues is needed. A regulatory and policy approach that is adaptable to the rapid advancements in AI technology is needed. This flexibility does not imply a relaxation of standards but rather a more dynamic and responsive regulatory process that can keep pace with AI innovation while upholding the highest standards of safety and efficacy. This review examines the regulatory and policy challenges associated with AI-enabled combination devices and explores potential ways of addressing those challenges.

## US regulatory landscape and challenges

The FD's 510(k) pathway is a major route for navigating the regulatory environment for medical devices in the United States, especially those that include artificial intelligence (AI). This route presents particular difficulties when it comes to AI-based medical devices, even while it helps new gadgets into the market. Because AI is inherently designed to learn and adapt over time, there are substantial regulatory barriers that must be overcome. New strategies are needed to guarantee both efficacy and safety. We acknowledge that FDA has other regulatory pathways for medical devices. The DeNovo classification process is used for novel devices without predicates that are low to moderate risk. The Premarket Approval (PMA) pathway is the most stringent and is required for Class III devices that support or sustain human life. However, the 510(k) pathway remains a major route for many AI-enabled devices.

While the 510(k) submission of medical devices requires new devices to be substantially equivalent to a lawfully marketed devices referred as “predicate device”, it was initially created for devices with static functionality. But the implementation of this criterion is made more difficult by A's dynamic learning capabilities, which enable post-market adjustments based on fresh data. The FDA is now investigating regulatory frameworks that allow AI algorithms to be improved iteratively while upholding strict safety and efficacy standards. The regulation of AI systems that are always upgrading is a considerable difficulty in this regard. Conventional regulatory frameworks are unable to manage the swift iteration cycle of artificial intelligence technologies. As a response, the FDA has taken into account a “predetermined change control plan” in its regulatory proposals, which permits manufacturers to alter AI algorithms after they have been approved without having to submit a new 510(k) application as long as the modifications stay within the parameters of the initial plan (7).

**Figure 1:**
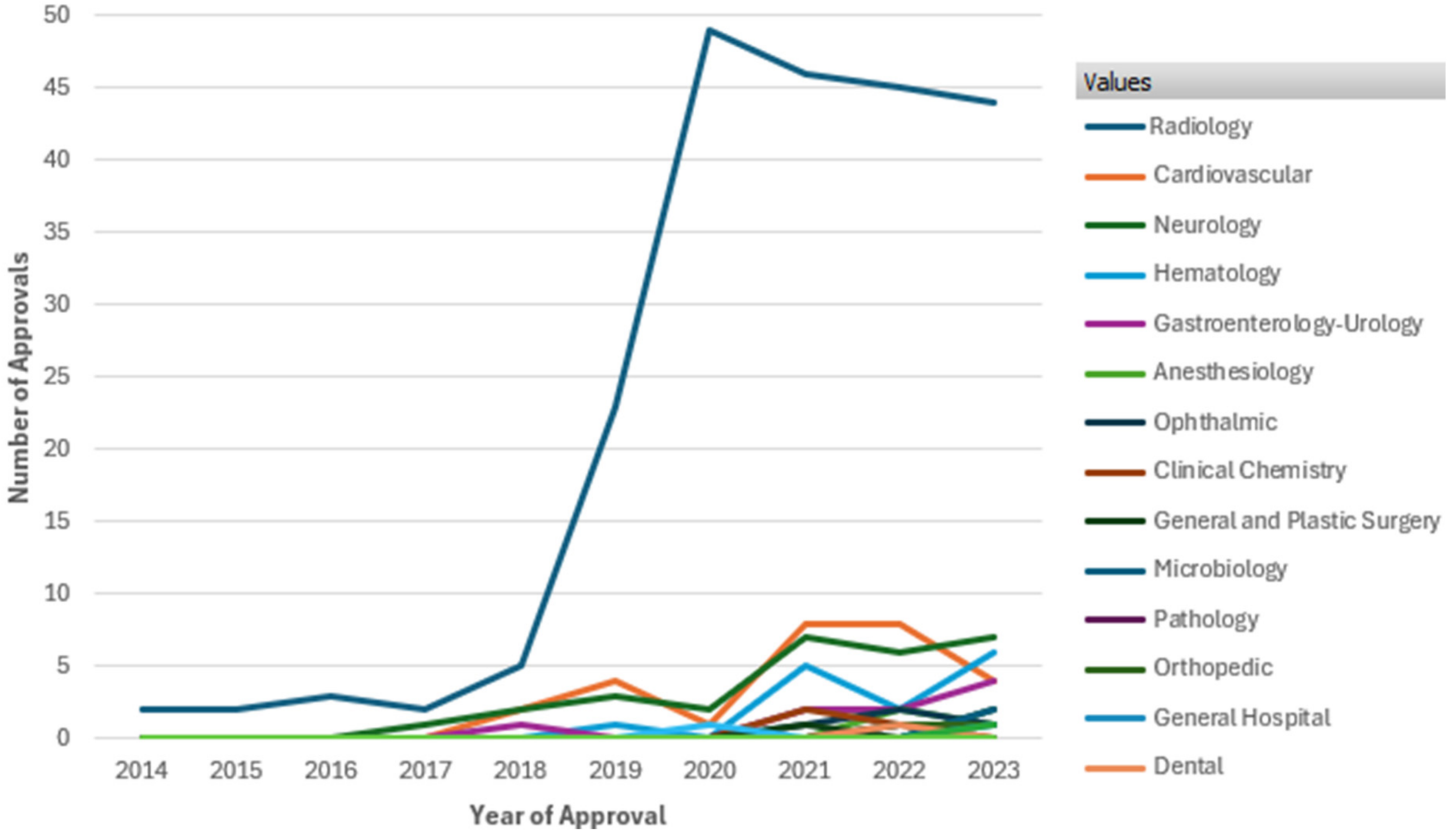
FDA approvals of AI/ML enabled devices by therapeutic area from 2014 to 2023. Data Source (8): U.S. Food and Drug Administration, Artificial intelligence and machine learning (AI/ML) enabled medical devices available online at https://www.fda.gov/medical-devices/software-medical-device-samd/artificial-intelligence-and-machine-learning-aiml-enabled-medical-devices, last accessed on Sept. 2024.

Figure 1 illustrates the number of AI/ML-enabled medical device approvals by FDA over the past decade. It highlights a significant overall increase in approvals starting in 2018, with a sharp rise peaking in 2020 at nearly 50 approvals. Radiology dominates the approvals, showing a steep increase from 2018 onward, far outpacing other therapeutic areas such as Cardiovascular, Neurology, Hematology, and Gastroenterology-Urology, which exhibit steady but smaller growth. This data underscores the growing integration and regulatory acceptance of AI/ML technologies in medical devices, especially in radiology (8).

Case studies and recalled devices provide important insights into the challenges associated with regulating AI in healthcare. The necessity of rigorous post-market surveillance and the probable need for recalibration of AI algorithms based on real-world outcomes are highlighted by the recall of an AI-based diagnostic tool, for instance, because of errors in real-world situations. These examples demonstrate the disparity that exists between the settings of clinical trials and real-world use, underscoring the need for regulatory frameworks that are both flexible and strict to monitor and manage these kinds of issues. While many AI/ML devices have been cleared through the 510(k) pathway without clinical trials, the FDA is increasingly focusing on real-world performance monitoring for these technologies.

Pre-market testing and post-market monitoring must be balanced to support innovation while maintaining patient safety within the new regulatory framework. The FD's adaptive regulatory strategy, which aims to keep up with technology changes, is demonstrated by its engagement with stakeholders through public workshops and guidance materials. AI-enabled medical device regulation presents special difficulties for the FD's 510(k) process, calling for a flexible and forward-thinking regulatory strategy. The FDA hopes to protect public health and promote innovation in AI by combining strong safety and efficacy measures with flexibility to accommodate A's iterative nature (9, 10).

Recently, the FDA rolled out the Emerging Drug Safety Technology Meeting (EDSTM) program which represents another strategic initiative by the regulating agency to engage with various stakeholders on the application of AI in drug safety. EDSTM program highlights the FD's proactive approach in facilitating dialogue and mutual learning, crucial for developing regulatory frameworks that can keep pace with technological advancements (11).

## European Union’s regulatory landscape and challenges

The Medical Device Regulation (MDR) of the European Union offers a framework for guaranteeing the effectiveness and safety of medical devices, including those that use artificial intelligence (AI). With the goal of enhancing clinical safety and facilitating the swift growth of technology in the healthcare industry, the MDR, which went into full force in May 2021, is a significant update from its predecessors (12).

There are major challenges with integrating AI into medical equipment, especially when it comes to conformance evaluations for adaptive algorithms. The dynamic nature of these algorithms necessitates a regulatory strategy that ensures patient safety while accommodating their evolution based on fresh data. In an effort to address these issues, the MDR places a greater focus on clinical evidence and post-market surveillance, requiring manufacturers to furnish thorough clinical data and to continuously track the effectiveness of their devices (13).

Predicting the behavior of adaptive AI algorithms as they learn from fresh data is one of the main issues in conformance assessments. The traditional regulatory methodology, which depends on fixed device features to determine compliance, is complicated by this unpredictability. In order to overcome this challenge, the MDR advocates for a risk-based strategy in which the degree of examination is commensurate with the possible risk that the device poses. The risk-based strategy adopted by regulatory bodies like the FDA and under the E's MDR, as outlined in their respective guidance documents, is a nuanced approach to device evaluation that tailors the intensity and scope of scrutiny based on the potential risk a device poses to patient safety (10, 12). In practice, this means that AI-enabled devices with higher risk profiles—such as those used in critical care settings or for making autonomous treatment decisions—undergo more rigorous examination. This may involve extensive clinical trials, thorough algorithm validation, and comprehensive post-market surveillance plans. Conversely, lower-risk AI applications, like those used for administrative tasks or non-critical decision support, may face less stringent review processes. The risk assessment for AI-enabled devices considers multiple factors, including the intended use and clinical context of the device, the level of autonomy of the AI system in decision-makin, the potential consequences of AI errors or malfunctions, the transparency and explainability of the AI algorithm and the quality and representativeness of the training data. In order to ensure that any modifications made by the AI do not jeopardize the devic's functionality or safety, this method requires manufacturers to put in place strong risk management and quality control procedures (14).

Overcoming regulatory barriers in the EU for medical devices with AI integration also entails resolving privacy and data protection issues. Strict guidelines are outlined in the General Data Protection Regulation (GDPR) for the handling of personal data, including health information that AI systems employ. Manufacturers of AI-integrated products must adhere to GDPR, which mandates open data processing procedures and the protection of data subjects’ rights (15).

## Regulatory challenges with AI-enabled medical devices

### FDA 510(k) pathway challenges

The IDx-DR case voices concern that the reliance of the FDA on predicate devices for the approval of AI-based technologies through the 510(k) pathway has its problems. Whereas this has been beneficial in fast-tracking the path to market approval for technologies considered “substantially equivalent” to already marketed devices, for an adaptive AI technology this process is cumbersome.

The fact that IDx-DR is able to continually learn and improve post-deployment, via updates from real-world experience, exposed huge challenges under a 510(k) framework predicated on static technologies. Again, this example will demonstrate how the static nature of the predicate-based process cannot coexist with an adaptive algorithm needing updates. This requires ongoing post-market surveillance and asks questions about the suitability of the 510(k) process for such dynamic technologies. This example demonstrates the intrinsic challenge of applying a traditional regulatory framework to AI technologies that are not “fixed” at the time of approval but evolve over time with growing risk for model drift or degradation (16–19). Table 1 summarized the challenges imposed by AI-enabled medical devices.

**Table 1:** Challenges in AI-enabled medical devices.

S.no.	Challenge	Description	Case study example
1	FDA 510(k) pathway challenges	Reliance on predicate devices for AI-based medical technologies, limiting the accommodation of adaptive AI algorithms.	IDx-DR (AI for Diabetic Retinopathy)
2	EU MDR conformity assessment challenges	Demonstrating safety and efficacy for adaptive AI algorithms under MDR is difficult due to their evolving nature.	HeartFlow FFRct (AI-based coronary diagnostics)
3	Post-market surveillance challenges	Continuous monitoring and updating of AI algorithms are required to ensure ongoing compliance and functionality.	Aidenc's veye lung nodules (AI for lung cancer detection)

### Challenges of EU MDR conformity assessment

The case of HeartFlow FFRct represents a very good example of how difficult the demonstration of long-term safety and efficacy of an AI algorithm is under EU MDR. Since the HeartFlow AI algorithm was of an evolving nature, this itself presented a different kind of challenge for the company because the expectations of the regulatory framework are that devices should not change after market approval.

The case underlines this challenge that adaptive AI poses for traditional conformity assessments. Because the algorithm was constantly changing, the clinical performance of the AI system could not be determined in a single point in time, as requested by the MDR. Instead, the learning capability of the AI system required constant updating and assessment, which does not fit well into the current regulatory framework of the EU. The case illustrates the more general problem of attempting to apply invariant regulatory standards to a technology which is itself continuously evolving and adapting, using a flexible and iterative approach to regulation (17, 20, 21).

### Challenges of post-market surveillance

The case of Aidenc's Veye Lung Nodule AI in detecting lung cancer nodules is just one of several examples that still call for post-market surveillance and monitoring of AI technologies. For as long as the AI system continues to learn from real-world data and new cases, its updating and monitoring on a continuous basis will be required to ensure its accuracy and effectiveness.

This case serves well to illustrate the complexity that arises in keeping adaptive AI algorithms stationary concerning their clinical performance over time. Other than most medical devices that stand still, AI systems, such as Veye, will inherently have to be updated after the market and recalibrated. Such ongoing surveillance poses significant regulatory challenges since the original approval does not account for these iterative learning processes. This case illustrates how post-market surveillance turns into an integral part of the life cycle of an AI device, which is necessary not only to stay compliant but also functional through their constant evolution (19).

## Addressing AI risks in healthcare

AI in healthcare refers to a variety of technological advancements that make it possible for Large Language Models (LL's) to carry out tasks like learning, problem-solving, and decision-making that normally need human intelligence. Effectively addressing AI threats in healthcare requires the development of strong frameworks. These frameworks should take sociological, legal, and ethical issues into account in addition to technological ones. Stakeholders in healthcare must work together to define rules unique to AI model creation, validation, and deployment, building on existing frameworks like the FD's Software Precertification (Pre-Cert) Program (22) and the European Unio's Ethics rules for Trustworthy AI (23) which foster innovative technologies while providing guidelines for lawful, ethical and robust development of trustworthy AI. To guarantee the dependability and safety of AI models throughout their lifecycle, these frameworks should place a strong emphasis on accountability, transparency, and ongoing model monitoring. An analysis of the Manufacturer and User Facility Device Experience (MAUDE) database from FDA reveals a total of 32 experiences for devices with Artificial Intelligence from 2021 to June 2024. Upon closer examination, none of the user experiences explicitly state AI as the cause of the device malfunction. In some cases such as an ablation catheter, AI was used for planning the AI-guided ablation while in others such as femoral stem, AI was used in creating a 3D model for total hip arthroplasty.

Over time, shifts in patient demographics, changes in medical procedures, or adjustments in data distribution can cause AI models used in healthcare to deteriorate. Preserving the efficacy of AI applications requires an understanding of and response to model degradation. Degradation problems can be quickly identified and fixed with the aid of ongoing monitoring, frequent updates, and feedback loops involving medical experts.

Model degradation is a significant challenge for AI-enabled medical devices, particularly those leveraging machine learning algorithms, as it can negatively impact both patient safety and the overall efficacy of the device. As these models are often trained on data collected from controlled environments, their performance can degrade over time when deployed in real-world settings. This degradation occurs primarily due to data drift, which refers to the changes in the statistical properties of input data after model deployment. Factors such as evolving patient demographics, shifts in disease prevalence, and modifications in clinical practices can all contribute to data drift, leading to less accurate predictions and potentially unsafe or ineffective medical outcomes. For example, predictive models initially designed for specific populations may underperform when applied to different patient groups with varying characteristics, which can increase the risk of misdiagnosis or inappropriate therapeutic decisions (24, 25).

Another key cause of model degradation is related to hardware and environmental changes that affect data acquisition. For instance, diagnostic devices relying on AI algorithms may encounter variations in sensor calibration, lighting, or other environmental conditions, leading to the collection of lower-quality input data. Such conditions can significantly reduce the accuracy of AI predictions, increasing the likelihood of device malfunction (26). This problem is exacerbated by the fact that AI models are dynamic and require continuous updates and recalibration to maintain performance over time. Regulatory bodies such as the FDA advocate for the implementation of robust post-market surveillance mechanisms, where the mode's real-world performance is continuously monitored, and updates are made in a controlled manner to address potential degradation issues before they can compromise patient safety.

The degradation of AI models also poses risks for long-term device efficacy, especially when clinical guidelines or treatment protocols evolve. A model trained under one set of standards may become obsolete when those standards change, rendering the device less effective. This phenomenon underscores the importance of adaptive regulatory frameworks that allow for timely updates and modifications to AI algorithms without compromising safety. The FD's guidance on Software as a Medical Device (SaMD) highlights the need for pre-specified change control protocols, which enable manufacturers to implement necessary changes while maintaining the devic's regulatory approval (27). Preemptive model assessment and improvement efforts can benefit from the lessons learnt from medical device recalls caused by unforeseen difficulties such as accuracy of medication list based on EM's (28).

These projects aim to identify points where bias can be introduced in the AI development lifecycle and explore ways to address it through risk management. This aligns with the need for preemptive model assessment and improvement efforts discussed earlier.

Additionally, the FDA plans to support initiatives that consider health inequities associated with AI use in medical product development, leveraging ongoing diversity, equity, and inclusion efforts. This approach complements the industr's efforts to reduce biases in AI applications through the use of representative and diverse datasets.

Furthermore, the FDA emphasizes the importance of ongoing monitoring of AI tools in medical product development. This focus on ensuring adherence to standards and maintaining performance and reliability throughout the AI lifecycle underscores the importance of continuous vigilance in addressing AI risks in healthcare. It reinforces the need for comprehensive testing standards and quick response systems to address unforeseen difficulties, as highlighted by the lessons learned from medical device recalls.

In healthcare AI faces a great deal of challenges due to data biases, which can result in disparities in treatment recommendations and diagnostic results. Together, the healthcare professionals and AI developers can detect and reduce biases in data collection, preprocessing, and model training since they understand how important unbiased data is. Reducing biases in AI applications can be achieved through the use of representative and diverse datasets and fairness-aware algorithms (29). Examining case studies pertaining to medical device recalls provides insightful information about the possible drawbacks of implementing insufficiently evaluated technologies in the healthcare industry. One example of a cautionary story about the significance of thorough testing and validation prior to widespread application is the recall of some imaging devices because of erroneous readings (30). Our recommendation is for AI stakeholders to establish comprehensive testing standards, post-market surveillance, and quick response systems to quickly address unforeseen difficulties by learning from such situations.

## Frameworks for adaptive policy

To be able to develop a framework for AI based CDs, it is necessary to understand how to evolve an inherently iterative system. For traditional devices, such as pacemakers and stents, changes or modifications once the device is marketed may require additional FDA review, either as a supplement to the premarket approval (PMA) or as a new 510(k) submission. It is unclear if this model is suitable for AI-based software, which is inherently iterative. For example, AI-based software can change its behavior in real time after it is distributed and has “learned” from clinical application and experience. This continuous learning could result in outputs that differ from what was initially reviewed prior to regulatory approval. Pre- market changes to devices may be important to monitor when related to physical devices but are not ideal for continuous software iterations (31).

## Principles of adaptive policy frameworks

Adaptive policy frameworks for medical devices need to account for principles such as transparency, accountability, collaboration and patient-centeredness (32, 33).

## Transparency

Ideally, AI -based software would be evaluated in prospective clinical trials using meaningful end points for patients. Rigorous pre- market assessment of the performance of AI- based software could also facilitate broader and faster access to these new technologies.

## Accountability

A risk assessment framework should be developed for the list of changes or software updates that would be considered safe v/s potentially risky given the implication of the changes. For example, if a software update could lead to potentially discharging a higher dose of drug upon administration so as to lead to an adverse reaction, then the parameters of the update and i's consequences need to be closely monitored and demonstrably validated through clinical studies (31).

## Collaboration

Collaboration between the FDA and device manufacturer is essential to evaluate the pre-market data and post marketing surveillance data in real time and make immediate risk based changes to the device. The recently launched Emerging Drug Safety Technology Meeting (EDSTM) program underscores the need for adaptive policy frameworks that can accommodate the iterative nature of AI technologies. By enabling continuous engagement between the FDA and industry stakeholders, the program supports the development of flexible regulatory approaches that balance innovation with patient safety. Recognizing these challenges, the FDA has recently outlined plans to support research that addresses critical aspects of AI performance in healthcare. According to a 2024 FDA paper, the agency intends to support demonstration projects focusing on several key areas (8).

## Patient-centeredness

Keeping the patient in mind is always the foremost goal of any combination device development. Policy considerations around patient centeredness need to focus on patient data privacy and consent. Data privacy must be respected and that patients should be adequately informed about what data is collected, how it is used, and who it is shared with, especially in systems that learn and adapt over time (34).

Privacy concerns are highlighted with the use of AI because the algorithms often require access to large quantities of patient data and may use the data in different ways over the time The location and ownership of servers and computers that store and access patient health information for healthcare AI to use are important in these scenarios. Regulation should require that patient data remain in the jurisdiction from which it is obtained, with few exceptions (35).

Another major challenge is the re-identification of patient data that is leaked in a privacy breach. These breaches have increased significantly over the past several years (36). Further to address the root cause, cybersecurity legislation needs to evolve in line with the latest cyber threats.

Currently, patients who sign up for one of the combination devices are often required to agree to online consent form (s) and most of them, in the paucity of time and knowledge, resort to just agreeing to the terms and conditions without comprehending how their data might be used (37). Policy to address patient consent needs to be carefully constructed around mandating that contracts clearly delineate the rights and obligations of the parties involved, and liability for the various potential negative outcomes.

## Conclusion

This article attempted to explore the integration of AI in CDs and its potential to revolutionize healthcare through improved functionality, accuracy, and adaptability. AI-enabled CDs offer opportunities for personalized treatment, real-time monitoring, and enhanced diagnostic capabilities. However, the adoption of these technologies also presents unique challenges in regulatory and policy frameworks, necessitating the evolution of these frameworks to address critical issues such as data privacy, algorithmic transparency, and patient safety. The FD's initiatives, such as EDSTM program, underscore the importance of continuous dialogue and adaptive regulatory approaches to keep pace with technological advancements. AI enabled device could gain benefit through EDSTM program that has facilitated discussions leading to the establishment of guidelines for CDs’ safe and effective use. By fostering collaboration among stakeholders and emphasizing a balanced approach between innovation and patient safeguards, the regulatory landscape can effectively support the safe and equitable adoption of AI-enabled combination devices. As technology continues to advance, it is essential to maintain a dynamic and forward-thinking approach to regulation, ensuring that the potential of AI in enhancing patient outcomes and advancing medical technology is fully realized.
